# Genotype-phenotype analysis of F-helix mutations at the kinase domain of *TGFBR2*, including a type 2 Marfan syndrome familial study

**Published:** 2012-01-11

**Authors:** Lin Zhang, Ling-Gen Gao, Ming Zhang, Xian-Liang Zhou

**Affiliations:** 1 Department of Cardiology, FuWai Hospital and Cardiovascular Institute, Chinese Academy of Medical Sciences and Peking Union Medical College, Beijing, China; 2 Department of Geriatric Cardiology, General Hospital of Chinese People's Liberation Army, Beijing, China; 3 Beijing Institute of Heart, Lung and blood vessel disease, AnZhen hospital, Beijing, China

## Abstract

**Purpose:**

Transforming growth factor beta receptor II (*TGFBR2*) gene mutations are associated with Marfan syndrome; however, the relationship between the mutations and clinical phenotypes are not clear.

**Methods:**

Genomic DNA from peripheral blood leukocytes of a Chinese proband with Marfan syndrome, five of the proband’s relatives, and 100 unrelated Chinese control subjects were isolated and screened for fibrillin-1 (*FBN1*) and *TGFBR2* gene mutations by direct sequencing, and a genotype-phenotype study was performed following a review of the literature on *TGFBR2* mutations in the search area. Also, the structure of TGFBR2 protein before and after gene mutation was analyzed.

**Results:**

The results identified a novel missense *TGFBR2* mutation p.V453E (c.1358T>A) in the proband and two relatives that was located in the F-helix in the kinase domain of TGFBR2. No such genetic change was observed in the unrelated controls. No *FBN1* mutation was detected in any of the subjects. Genotype-phenotype analyses indicated that F-helix mutations are related to type 2 Marfan syndrome and Loeys-Dietz syndrome, and these mutations can lead to severe cardiovascular (93.8%) and skeletal (81.3%) lesions and minor ocular lesions (25%). Losartan treatment can slow-down the progression of aortic lesions.

**Conclusions:**

The ﬁndings extend the mutation spectrum of Marfan syndrome, and that mutations at the F-helix in the kinase domain of TGFBR2 may be associated with the development of severe cardiovascular and skeletal lesions and minor ocular lesions. These findings have implications for genetic testing, diagnosis, and treatment in individuals with transforming growth factor beta (TGF-β) signaling-related disorders.

## Introduction

Marfan syndrome (MFS) is an autosomal dominant extracellular connective tissue disorder that is clinically diagnosed according to the Ghent criteria [1]. Classic MFS presents as abnormal features in skeletal, ocular, and cardiovascular systems. The skeletal abnormalities of affected individuals typically include tall stature, long slender limbs (dolichostenomelia), scoliosis, arachnodactyly, and pectus excavatum or carinatum. About 80% of MFS sufferers have ectopia lentis, which is almost always bilateral. Progressive dilatation of the aorta is the main cardiovascular irregularity of MFS individuals, and acute aortic dissection originating from dilation of the ascending aorta is the leading cause of premature death in untreated MFS individuals [2]. Other manifestations, including spontaneous pneumothorax, apical blebs, striae atrophicae, and lumbosacral dural ectasia, can also be observed in MFS patients [3,4].

MFS is most commonly caused by fibrillin-1 (*FBN1*) gene mutations at chromosome 15q21.1, with the mutation types being point mutations, insertions, large and small deletions, and splice mutations. The mutations are spread throughout almost the entire *FBN1* gene without obvious predilection for any given region. Recent studies show that genetic heterogeneity exists in MFS individuals, and transforming growth factor beta receptor II (*TGFBR2*) gene mutations have been identified in a subset of patients with MFS – which was termed as type 2 Marfan syndrome (MFS2) [5]. Mutations in *TGFBR2* have also recently been identiﬁed in patients with Loeys-Dietz syndrome, and familial thoracic aortic aneurysms [6,7].

Transforming growth factor beta superfamily signaling is of great importance in the maintenance of the extracellular matrix and tissue homeostasis [8]. One important member of this gene superfamily is *TGFBR2*, which encodes for the trans-membrane kinase receptor involved in signal transduction of the transforming growth factor beta (TGF-β) family of ligands [9]. This gene is composed of seven exons encoding 567 amino acids to form an NH_2_-terminal ligand binding domain, a trans-membrane region, and a constitutively active COOH-terminal serine/threonine kinase domain [10]. These structures are critical for TGF-β signaling, which plays a key role in normal extracellular connective tissue functions. Several *TGFBR2* mutations have been reported in association with vascular abnormalities [5,11-13], with most being missense substitutions or nonsense mutations in the penultimate or ﬁnal exons and predicted to disrupt the kinase domain. At present no strict genotype-phenotype correlations between *TGFBR2* mutations and MFS2 have been established, and great clinical variability of cardiovascular, skeletal, and ocular system manifestations exists among *TGFBR2* mutation carriers.

This study describes the genetic and clinical features of a Chinese family, the proband of which displays the MFS2 phenotype genetically caused by mutation at the *TGFBR2* locus. A literature review was also undertaken with the aim of exploring the relationship between mutations at the region in this study and clinical phenotypes.

## Methods

### Study subjects

This study was conducted in accordance with the Declaration of Helsinki and approved by the Fuwai Cardiovascular Center Ethics Committee.

An MFS patient and family and 100 unrelated controls participated in this study. All subjects were of Han Chinese origin. Written informed consent was obtained from all subjects (or their parents if the subject was less than 18 years of age).

**Figure 1:**
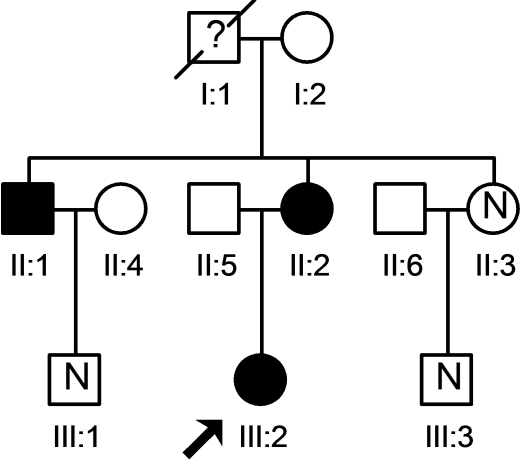
Family pedigree. The proband (III: 2, mutation carrier) presented with an ascending aortic aneurysm and underwent a ROSS procedure. Both the proband’s mother (II: 1) and uncle (II: 2) carry a *TGFBR2* mutation and have cardiovascular and skeletal system irregularities. The proband’s grandfather (I: 1) died suddenly at 43 years of age from an ascending aortic dissection; however, no genetic test was performed. No mutation was found in III: 1 and III: 3 who have normal phenotypes.

The pedigree of the enrolled family is presented in Figure 1. The proband of the enrolled family was a 12-year-old female (III: 2, in Figure 1), who presented for examination of her cardiovascular system because previous X-ray results indicated aortic root dilation. Other family members (II: 1, II: 2, II: 3, III: 1, III: 3, in Figure 1) of the proband were recruited and underwent multidisciplinary clinical and noninvasive instrumental studies. Our assessments covered all the diagnostic criteria for MFS [5]. A physical examination and echocardiography were performed on the unrelated control subjects to exclude MFS and other congenital abnormalities. The aortic root diameter was evaluated by echocardiography.

### Genetic analysis

Genomic DNA was isolated from peripheral venous blood leukocytes of all subjects using a commercially available kit (Qiagen Inc., Germantown, MD). Following amplification by polymerase chain reaction (35 cycles), mutation screening (which allowed for the scanning of 65 *FBN1* exons and flanking regions, including the splice sites up to the branching regions [primers used for bidirectional sequencing are presented in Appendix 1]) was performed using an ABI3730xl Sequencer (Applied Biosystems, Foster City, CA). The primers used for bidirectional sequencing of *TGFBR2* are presented in Table 1. *TGFBR2* mutation screening of all seven encoding exons was performed after finding that all the exon-intron boundaries of *FBN1* were negative. Mutation numbering refers to the *TGFBR2* cDNA GenBank reference sequence: NM_003242.5, with the A of the ATG translation initiation codon as nucleotide +1.

**Table 1:** Primers used for the amplification of *TGFBR2*.

**Exon**	**Forward primer ( 5’→ 3’)**	**Reverse primer ( 5’→ 3’)**	**Melting temperature (°C)**	**Product length (bp)**
Exon1	AGCTGTTGGCGAGGAGTTTCCTGTTT	GCCGCCTTCAGATAACCAACTTCTCAAAC	58	765
Exon2	TTGACAAAAGCAAATGGCTACTC	GGAAAGGGAAATGGAACAGG	58	539
Exon3	AAACAGAAAAGAGTAGAAAAGCATAGG	TGATGAGAACCAGAACAAACCCT	58	574
Exon4	AGCAGGGGATGACGAACAGA	GAAGGATTTGAAGATAGGAGGAGAA	58	1226
Exon5	TGTTTATGGTCTTTAGAGGGTTTTC	CCAAATAGTTCTGGGATGGTTGTA	58	507
Exon6	AAAACCTAAGCTCCGTGAC	TTAACAGGGCCATAGAACA	58	545
Exon7	GTGTTGGGAGTGTTAGTGTA	CAATGTCAAAGGCATAGAAT	58	695

Sequences are given in the 5’→ 3’direction. *TGFBR2*: transforming growth factor beta receptor II gene.

### In silico prediction of the effects caused by TGFBR2 mutations

The human TGFBR2 reference sequence accession NP_003233.4 was used to perform in silico prediction, and the SWISS-MODEL tool was used [14,15]. Mutagenesis and visualization were performed to determine the possible effects of the mutation on the structure of their corresponding modules during in silico 3D modeling analysis.

### Genotype-phenotype investigation

A literature search of *TGFBR2* mutations and related clinical manifestations was performed and mutations were classified according to their locations in the gene and functional domains. Correlations between mutations at the region where the mutation in this study was found and their clinical features were analyzed.

### Statistical analysis

Statistical analyses were performed using SPSS software version 15.0 (SPSS Inc., Chicago, IL). Phenotypes detected which involved separate organ systems were compared with the Pearson χ^2^ test; or using a Fisher’s exact test for small samples. All the reported p values are two-sided, and a p value of <0.05 was considered significant.

## Results

### Clinical features of the patients

The clinical manifestations of all family members are summarized in Table 2. The proband was confirmed as an MFS patient [5], and a familial history suggestive of MFS was reported. A chest computed tomography of the proband revealed that she had an ascending aortic aneurysm, and echocardiography showed an aortic root diameter of 50 mm. The patient underwent a ROSS procedure (graft replacement of the ascending aorta) to treat her aortic aneurysm. A characteristic habitus with tall stature, long slender limbs (dolichostenomelia) was observed. She was 168 cm tall at 12 years of age, and her arm span to height ratio was 1.15. Pectus carinatum, scoliosis (25°), dolichostenomelia, arachnodactyly, and positive wrist and thumb signs were also noted; the findings indicated that her skeletal system was involved. Striae atrophicae was also found.

**Table 2:** Clinical manifestation of the proband and her family members.

**Parameters**	**III:2**	**II:2**	**II:1**	**I:1**
Age	12	35	39	Death at 43
Sex	Female	Female	Male	Male
Cardiovascular system	Aortic root dilation (aortic root diameter=50 mm); Aortic regurgitation; Mitral valve regurgitation; Dilatation of the main pulmonary artery.	Aortic root dilation (aortic root diameter=45 mm); Aortic regurgitation.	Aortic root dilation (aortic root diameter=48 mm)	Aortic root dilation; Dissection of the ascending aorta
Skeletal system	Arm span to height ratio=1.15; Wrist and thumb signs; Scoliosis of=25°; Pectus excavatum; Dolichostenomelia; Arachnodactyly.	Arm span to height ratio=1.09; Dolichostenomelia; Arachnodactyly; Wrist and thumb signs.	Arm span to height ratio=1.05; Dolichostenomelia; Arachnodactyly; Joint hypermobility.	?
Ocular system	Nil	Nil	Nil	?
Pulmonary system	Nil	Nil	Nil	?
Skin and integument	Striae atrophicae	Nil	Nil	?
Ghent nosology	Fulflled	Not fulflled	Not fulflled	?
*TGFBR2* mutation	c.1358T>A, p.V453E	c.1358T>A, p.V453E	c.1358T>A, p.V453E	?

The proband’s mother had a characteristic habitus with tall stature and long slender limbs (arm span to height ratio was 1.09); dolichostenomelia, arachnodactyly, and wrist and thumb signs were noted; and dilatation of the ascending aorta (aortic root diameter was 45 mm) and aortic regurgitation were observed.

No ocular lesions were found in either the proband or her mother.

Further findings positive for MFS in other family members are presented in Table 2. No positive findings were detected in the control subjects.

Losartan (25 mg per day) was used as previously reported [16] in the proband after surgery to prevent aortic enlargement and associated cardiovascular pathologic changes, and no recurrent aortic lesion was found after losartan treatment. Losartan (50 mg per day) was also used in the other individuals (II: 1 and II: 2) who had *TGFBR2* mutations. No obvious progression of aortic root dilation was detected after losartan therapy.

### Mutation analysis

**Figure 2:**
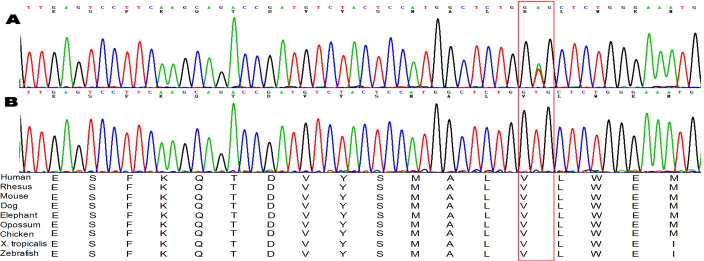
Sequencing plots of *TGFBR2*. A novel heterozygous missense mutation of *TGFBR2* (c.1358T>A, p.V453E) affecting an evolutionarily conserved amino acid was detected in the proband (III: 2), her mother (II: 1) and uncle (II: 2). The mutation was absent in other family members (III: 1, III: 3, II: 3) and the controls.

A novel missense mutation (p.V453E (c.1358T>A) in exon 5) was identified in *TGFBR2* in the proband (III: 2), the proband’s mother (II: 2), and the proband’s uncle (II: 1; see Figure 2). This alteration was not detected in the 100 unrelated chromosomes from ethnically matched Han Chinese controls.

The mutation identified affects a highly conserved nucleotide and is predicted as damaging protein production or affecting protein function. The mutation located in the F-helix in the kinase domain of TGFBR2, which was formed by the 440–457 residues encoded by the 1318–1370 base pairs.

No *FBN1* mutation was found in any of the subjects of the study.

### Protein structure changes due to mutations

**Figure 3:**
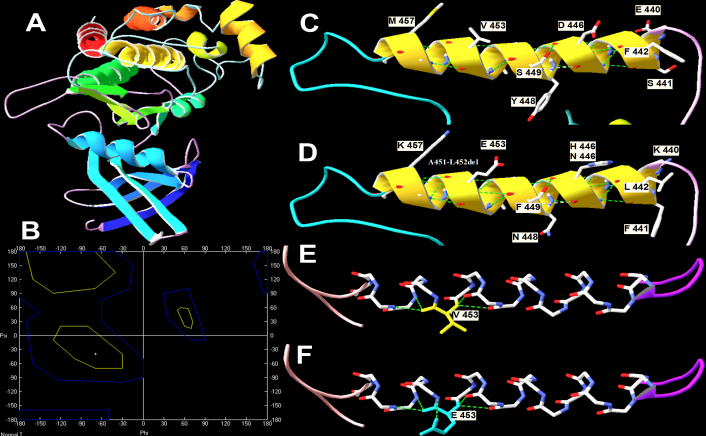
Prediction of the impact of mutations at the F-helix in the kinase domain of TGFBR2. **A**: Three-dimensional plot of normal TGFBR2 drawn with Swiss-Pdb Viewer. **B**: Ramachandran plot of predicted p.V453E. Phi and Psi angles changed after mutation. **C** and **D**: The normal and mutant residues at this region. **E** and **F**: The structure change after the normal hydrophobic valine residue was replaced the by a hydrophilic glutamic acid.

The missense mutation involves a highly conserved valine residue that is located in the F-helix in the kinase domain of TGFBR2. The F-helix in the kinase domain plays an important role in maintaining the function of TGFBR2. The mutant residue is predicted to induce steric clashes with its surroundings. In silico modeling analysis showed that four additional hydrogen bonds were formed; and the conformation changed after the valine residue was replaced by glutamic acid (Figure 3E,F). This structural alteration may perturb the crucial F-helix structural components of the protein kinase domain. Previously reported mutations at the F-helix can alter the protein kinase domain of TGFBR2 to impair the TGFBR2 pathway (Figure 3C,D), and can therefore lead to MFS or MFS-like phenotypes [17].

### Genotype-phenotype relationships

The results of the literature review of the F-helix of the kinase domain of TGFBR2 are shown in Table 3. Nine kinds of mutations (all of which are point mutations, except for one deletion change) were found in this domain, as reported in 16 studies [5,12,13,18-25]. Almost all mutation carriers (15/16, 93.8%) displayed major cardiovascular system abnormalities. Four cases (4/16, 25%) were reported as showing minor ocular lesions, and no ectopia lentis was reported. Skeletal system abnormalities were reported in 81.3% (13/16) of cases, and 31.3% (5/16) had major skeletal system lesions. The cardiovascular and skeletal system had significantly higher involvement rates when compared to the ocular system (p<0.01 of both compares), but no significant difference in involvement rates was found between the cardiovascular system and the skeletal system (93.8% versus 81.3%, p=0.60).

**Table 3:** Phenotypes caused by genetic changes at F-helix in the kinase domain of *TGFBR2*.

**Reference**	**Nucleotide**	**Protein**	**Diagnosis**	**CS**	**OS**	**SS**	**S**	**LDS specific symptoms**	**Age (years)**	**Sex**	**Ghent nosology**
[12]	c.1318G>A	p.E440K	LDS	M	Strabismus	m	-	Involve	9	Male	Not fulfilled
[22]	c.1322C>T	p.S441F	MFS2	M	Myopia	M	m	Not involve	14	Male	Fulfilled
[19]	c.1324T>C	p.F442L	LDS	M	-	m	m	Involve	36	Female	Not fulfilled
[21]	c.1336G>A	p.D446H	LDS	M	Blue sclerae	m	-	Involve	1	Male	Not fulfilled
[23]	c.1336G>C	p.D446H	LDS	M	-	-	-	Involve	7	Female	Not fulfilled
[13]	c.1336G>A	p.D446H	LDS	M	Blue sclerae; Strabismus	m	-	Involve	-	Male	Not fulfilled
[18]	c.1336G>C	p.D446N	MFS2	M	-	m	-	Not involve	4	Female	Not fulfilled
[23]	c.1336G>A	p.D446N	LDS	M	-	m	m	Involve	26	Female	Not fulfilled
[25]	c.1342T>A	p.Y448N	LDS	?	?	?	?	Involve	?	?	Not fulfilled
[5]	c.1346C>T	p.S449F	MFS2	M	-	M	-	Not involve	12	Female	Not fulflled
[25]	c.1346C>T	p.S449F	LDS	M	-	m	m	Involve	neonatal	?	Not fulfilled
[23]	c.1346C>T	p.S449F	MFS2	M	-	M	-	Involve	13	Female	Not fulfilled
This study	c.1358T>A	p.V453E	MFS2	M	-	M	m	Not involve	12	Female	Fulfilled
[24]	c.1370T>A	p.M457K	MFS2	M	-	m	m	Not involve	9	Female	Not fulfilled
[20]	c.1370T>A	p.M457K	LDS	M	-	M	-	Involve	neonatal	Male	Not fulfilled
[23]	c.1351_1356del	p.A451_L452del	LDS	M	-	-	-	Involve	1	Male	Not fulfilled

M: major criterion fulfilled, m: minor criterion fulfilled, -: organ system not involved, ?: not mentioned. CS: Cardiac system, OS: Ocular system, SS: Skeletal system, S: Skin and integument. LDS: Loeys Dietz Syndrome, MFS: Marfan Syndrome. Nucleotide numbering reflects cDNA numbering with +1 corresponding to the A of the ATG translation initiation codon in the reference sequences (*TGFBR2*: NM_003242.5). The initiation codon is codon 1.

MFS2 was reported in 37.5% (6/16 cases) of all the patients, but only 12.5% (2/16 cases) fulfilled the Ghent nosology criteria. The remaining 62.5% are Loeys-Dietz Syndrome individuals. The occurrence rates of MFS2 and Loeys-Dietz Syndrome caused by mutations in this region were comparable (p=0.29). Mutations in this region tend to attack the carriers before adulthood, and a significant higher illness detection rate was found in patients <16 years old (p=0.012).

## Discussion

Marfan syndrome is an autosomal dominant connective tissue disorder involving multiple organ systems. Patients with young-onset cardiovascular lesions failing to meet the Ghent criteria have been classiﬁed as Marfan-like connective tissue disorders. After the first report by Boileau et al. [26] in 1993, a Marfan-like phenotype that was not linked to the *FBN1* mutation was subsequently designated Marfan syndrome type 2 (MFS2). Diagnosis of MFS2 consists of severe cardiovascular ﬁndings, which can include the sudden death of affected people at a young age owing to a thoracic aortic dissection, and typical MFS skeletal features, but ocular ﬁndings were rare [27]. Genetic analysis of MFS2 patients by Mizuguchi et al. [5] identified a de novo chromosomal rearrangement involving chromosome 3p24.1 that can disrupt *TGFBR2* function to result in protein truncation due to abnormal splicing. Further studies demonstrate that the MFS2 phenotype is caused by mutations in *TGFBR2*.

TGF-β family cytokines form a complex signaling pathway and control several cellular processes, including cell proliferation and differentiation, apoptosis, and extracellular matrix formation [28]. Incorrect TGF-β signaling may lead to a decreased and disordered incorporation of fibrillin into the connective tissue matrix, and this abnormality in turn results in human MFS phenotypes [29]. Two types of trans-membrane receptors, type I (TbRI) and type II (TbRII), transduce the signals of TGF-β into the cell. TbRI and TbRII are encoded by *TGFBR1* and *TGFBR2*, respectively [30,31]. The identification of *TGFBR2* mutations in patients with MFS2 provided the ﬁrst direct link between a human connective tissue disorder and abnormal TGF-β signaling. TGFBR2 mutations may cause a loss in function of TbRII and lead to MFS2, Loeys-Dietz Syndrome [32] and Type 2 Thoracic aortic aneurysms and dissections.

In the present study we directly sequenced the entire coding region of *FBN1* and *TGFBR2*, and found a previously unreported *TGFBR2* mutation, p.V453E (c.1358T>A). V453 is a highly conserved residue located in the F-helix in the kinase domain of TbRII. The F-helix functions as a scaffold to correctly position catalytic, substrate-binding, and substrate residues [33]. The D-helix in the protein kinase domain is either close to or makes direct contact with the substrate. Mutations at the F-helix will perturb F-helix-D-helix communication and impair TGFBR2 signaling. In this study, the V453E mutation replaced the hydrophobic valine with a hydrophilic glutamic acid to form more hydrogen bonds, which altered the conformation of the F-helix (Figure 3E,F). This alteration destabilized the F-helix, which in turn perturbs the protein structure and function of TGFBR2. Therefore, the newly identified p.V453E missense mutation could seriously disturb the TGF-β signaling pathway, the consequence of which is MFS2 phenotypes in affected individuals. These findings aid in understanding that MFS can be caused not only by mutations in *FBN1* but also by mutations in *TGFBR2*.

More research is needed to better understand the relationship between *TGFBR2* mutations and MFS2 phenotypes. Great clinical heterogeneity was observed among *TGFBR2* mutations carriers [34]. In the present study, we found that F-helix mutation carriers had a very high major cardiovascular system involvement rate (93.8%). TGF-β signaling drives aneurysm progression in MFS. Habashi et al. [35] and Holm et al. [36] found that an angiotensin II receptor blocker, losartan, can inhibit TGF-β-mediated activation of extracellular signal–regulated kinase. Therefore, losartan can be widely used to treat patients with MFS. In our study, no obvious progression of aortic lesion was observed in all of the affected individuals after losartan treatment.

The proband with a *TGFBR2* mutation fulfilled the current diagnostic criteria of MFS2 [22], indicating an association between the genotype of V453E alterations and MFS2. Nine kinds of mutations at the F-helix of TGFBR2 have been reported, with residue sites 446 and 449 having higher mutation frequencies. Patients with mutations at this region are more prone to severe cardiovascular events and skeletal lesions (Table 3). Because mutations at the F-helix can seriously impair the TGF-β signaling pathway, individuals who have mutations at this region display a higher ratio (p=0.012) of developing severe clinical manifestations before adulthood.

Previous studies indicate that ectopia lentis is rare in MFS2 individuals [5,18], and a study performed in Chinese patients with Marfan-related disorders also found no ocular involvement [32]. In the Hilhorst-Hofstee et al. [37] study, they thought that ectopia lentis is caused by the lower production of ﬁbrillin-1 and not by perturbation of the TGF-β signaling. Consistent with these results, no ocular involvement was observed in patients of our study who had *TGFBR2* mutations, and no ectopia lentis was found in patients carrying a mutation at the F-helix. Only four F-helix mutation carriers (4/16, 25%) were reported as showing minor ocular lesions. One MFS2 patients had myopia, and in the three patients with Loeys-Dietz Syndrome, two blue sclerae and two strabismus were reported (Table 3).

In conclusion, this study reports a novel *TGFBR2* mutation in a Chinese family with a MFS2 proband. Mutations at the F-helix in the kinase domain of *TGFBR2* are prone to developing severe cardiovascular phenotypes and skeletal irregularities. Losartan can slow-down the progression of aortic lesion. Mutations in this region are also related to young-onset clinical phenotypes. Minor ocular abnormalities can be found in a low percentage of carriers with mutation at the F-helix. These findings have implications for genetic testing, diagnosis, and treatment in individuals with MFS2, and other TGF-β signaling-related disorders.

## Appendix 1

Primers used for the amplification of *FBN1*. To access the data, click or select the words “Appendix 1.” This will initiate the download of a compressed (pdf) archive that contains the file.
